# Supplementary material for: A billion years arms-race between viruses, virophages, and eukaryotes
Source: eLife. 2023 Jun 26;12:RP86617. doi: 10.7554/eLife.86617 (PMC10328495; doi:10.7554/eLife.86617)
Supplement: Supplementary file 1. [file elife-86617-supp1.docx]

**Supplementary file 1.** Viral species included in the final multiple sequence alignments (54 taxa) with their accession numbers and source reference.

| **Species** | **ID** | **GenBank/metagenomic accessions** | **Source** |
| --- | --- | --- | --- |
| *Maverick* (*Danio rerio*) | Proteo5 | NC_007136.7: 27671708-27687993 | (Barreat and Katzourakis, 2021) |
| *Maverick* (*Xenopus tropicalis*) | Proteo18 | NC_030686.1: 6013889-6026611 | (Barreat and Katzourakis, 2021) |
| *Maverick* (*Anolis carolinensis*) | Proteo2 | NC_014778.1: 129158247-129172346 | (Barreat and Katzourakis, 2021) |
| *Maverick* (*Xiphophorus hellerii*) | Proteo19 | QPIH01000028.1: 6019497-6036410 | (Barreat and Katzourakis, 2021) |
| *Maverick* (*Oreochromis niloticus*) | Proteo15 | NW_020327416.1: 45576-61017 | (Barreat and Katzourakis, 2021) |
| *Maverick* (*Oxygymnocypris stewartii*) | Proteo11 | QVTF01001200.1: 1425766-1443135 | (Barreat and Katzourakis, 2021) |
| *Maverick* (*Plutella xylostella*) | Group2_9 | NW_011952032.1: 283307-321039 | This work |
| *Maverick* (*Ostrinia furnacalis*) | Group2_10 | NW_021132744.1: 18414-41178 | This work |
| *Maverick* (*Diachasma alloeum*) | Group2_5 | NW_021681489.1: 105940-131438 | This work |
| *Maverick* (*Diachasma alloeum*) | Group2_6 | NW_021680987.1: 30857-54497 | This work |
| *Maverick* (*Photinus pyralis*) | Group2_7 | NW_022171288.1: 44859-60568 | This work |
| *Maverick* (*Sitophilus oryzae*) | Group2_8 | NW_022147237.1: 411147-463473 | This work |
| Metagenomic NCLDV | NCLDV_Roux3157 | Trout_Epilimnion_TBL_comb48_EPIDRAFT_1006965 | (Roux et al., 2017) |
| Metagenomic NCLDV | NCLDV_Mo545 | SRX331950.48.dc.fa_4042 | (Moniruzzaman et al., 2020) |
| Metagenomic NCLDV | NCLDV_Mo338 | SRX330942.38.dc.fa_3117 | (Moniruzzaman et al., 2020) |
| Metagenomic NCLDV | NCLDV_Mo338 | SRX330942.38.dc.fa_13478 | (Moniruzzaman et al., 2020) |
| Metagenomic NCLDV | NCLDV_Roux1456 | Mendota_contig-68000307_4926_nucleotides | (Roux et al., 2017) |
| Metagenomic NCLDV | NCLDV_Sch668 | MN740268.1 | (Schulz et al., 2020) |
| *Shrimp hemocyte iridescent virus* | — | NC_055165.1 | NCBI |
| *Infectious spleen and kidney necrosis virus* | — | NC_003494.1 | NCBI |
| *Heterosigma akashiwo virus 01* | — | NC_038553.1 | NCBI |
| *Akhmeta virus* | — | NC_055230.1 | NCBI |
| *Tupanvirus soda lake* | — | KY523104.2 | NCBI |
| *Diadromus pulchellus ascovirus 4a* | — | NC_011335.1 | NCBI |
| Metagenomic PLV | PLV_BS940 | 3300009435_____Ga0115546_1002426 | (Bellas and Sommaruga, 2021) |
| Metagenomic PLV | PLV_BS718 | Ga0115028_10000066 | (Bellas and Sommaruga, 2021) |
| Metagenomic PLV | PLV_BS13 | V563K_contig_8723_len_16581_bp | (Bellas and Sommaruga, 2021) |
| Metagenomic PLV | PLV_BS395 | ERR1823950_NODE_975_length_11499_cov_287.344855 | (Bellas and Sommaruga, 2021) |
| Metagenomic PLV | PLV_BS539 | ERX2821583_NODE_576_length_19830_cov_33.560977 | (Bellas and Sommaruga, 2021) |
| Metagenomic PLV | PLV_BS262 | HanCross_NODE_1742_length_13974_cov_48.888145 | (Bellas and Sommaruga, 2021) |
| Metagenomic PLV | Plike_4 | SAF4 | (Yutin et al., 2015) |
| Metagenomic PLV | Plike_7 | INO1 | (Yutin et al., 2015) |
| Metagenomic PLV | Plike_17 | RED1 | (Yutin et al., 2015) |
| Metagenomic PLV | Plike_1 | SAF1 | (Yutin et al., 2015) |
| Metagenomic PLV | Plike_19 | YSL1 | (Yutin et al., 2015) |
| Metagenomic PLV | Plike_25 | ACE1 | (Yutin et al., 2015) |
| Metagenomic virophage | Virophage_PE125 | 3300009070_____Ga0066256_1001637 | (Paez-Espino et al., 2019) |
| Metagenomic virophage | Virophage_PE85 | 3300009781_____Ga0116178_10003357 | (Paez-Espino et al., 2019) |
| Metagenomic virophage | Virophage_PE47 | 3300012984_____Ga0164309_10000286 | (Paez-Espino et al., 2019) |
| Metagenomic virophage | Virophage_PE169 | 3300009154_____Ga0114963_10000678 | (Paez-Espino et al., 2019) |
| Metagenomic virophage | Virophage_PE184 | 3300007609_____Ga0102945_1000484 | (Paez-Espino et al., 2019) |
| Metagenomic virophage | Virophage_Roux27 | TBH_10005622 | (Roux et al., 2017) |
| *Maverick*-related virus strain Spezl | — | NC_015230.1 | NCBI |
| Yellowstone Lake virophage 7 | — | KM502591.1 | NCBI |
| Zamilon virus | — | NC_022990.1 | NCBI |
| Yellowstone Lake virophage 5 | — | NC_028269.1 | NCBI |
| Dishui Lake virophage 6 | — | MN940573.1 | NCBI |
| Dishui Lake virophage 2 | — | MN940570.1 | NCBI |
| *Bat mastadenovirus WIV17* | — | NC_034626.1 | NCBI |
| *Murine mastadenovirus A* | — | AC_000012.1 | NCBI |
| *Murine adenovirus 2* | — | NC_014899.1 | NCBI |
| *Fowl aviadenovirus 5* | — | NC_021221.1 | NCBI |
| *Frog adenovirus 1* | — | NC_002501.1 | NCBI |
| *Snake adenovirus 1* | — | NC_009989.1 | NCBI |
