# Supplementary material for: A billion years arms-race between viruses, virophages, and eukaryotes
Source: eLife. 2023 Jun 26;12:RP86617. doi: 10.7554/eLife.86617 (PMC10328495; doi:10.7554/eLife.86617)
Supplement: Supplementary file 2. — The best supported position of the root was on the branch leading to virophages (53.9%), followed by NCLDVs and metagenomic PLV BS539 (27.4%). Other root positions received <6% support. The frequencies of trees with a certain position of the root were estimated by filtering different topologies in PAUP. Number of generations = 140 million. [file elife-86617-supp2.docx]

**Supplementary file 2.** Distribution of root positions calculated from the MCMC posterior tree sample. The best supported position of the root was on the branch leading to virophages (53.9%), followed by NCLDVs and metagenomic PLV BS539 (27.4%). Other root positions received < 6% support. The frequencies of trees with a certain position of the root were estimated by filtering different topologies in PAUP. Number of generations = 140 million.

| **Root position** | **Number of trees** | **Percentage** | **Cumulative sum** |
| --- | --- | --- | --- |
| **Virophages** | **15099** | **53.923** | **53.923** |
| NCLDVs + PLV_BS539 | 7672 | 27.399 | 81.322 |
| PLV_BS539 | 1572 | 5.614 | 86.936 |
| NCLDVs | 1489 | 5.318 | 92.254 |
| Virophages + NCLDVs + PLV_BS539 | 1388 | 4.957 | 97.211 |
| PLV_BS13 | 185 | 0.661 | 97.872 |
| Virophages + PLV_BS13 | 159 | 0.568 | 98.439 |
| Adenoviruses | 107 | 0.382 | 98.821 |
| Virophages + NCLDVs | 44 | 0.157 | 98.979 |
| Virophages + NCLDVs + PLV_BS539 + PLV_BS13 | 35 | 0.125 | 99.104 |
| Adenovirus + NCLDVs + PLV_BS539 | 29 | 0.104 | 99.207 |
| Adenoviruses + NCLDVs | 4 | 0.014 | 99.221 |
| Virophages + Adenoviruses | 3 | 0.011 | 99.232 |
| NCLDVs + PLV_BS539 + most PLVs | 1 | 0.004 | 99.236 |
| Virophages + PLV_BS13 + Adenoviruses | 1 | 0.004 | 99.239 |
| Adenovirus + PLV_BS13 | 1 | 0.004 | 99.243 |
| most PLVs | 0 | 0.000 | 99.243 |
| *Mavericks 1* + *Mavericks 2* | 0 | 0.000 | 99.243 |
| *Mavericks 1* | 0 | 0.000 | 99.243 |
| *Mavericks 2* | 0 | 0.000 | 99.243 |
| Virophages + most PLVs | 0 | 0.000 | 99.243 |
| Adenoviruses + most PLVs | 0 | 0.000 | 99.243 |
| *Mavericks 1* + *Mavericks 2* + PLV_BS13 | 0 | 0.000 | 99.243 |
| *Mavericks 1* + *Mavericks 2* + Adenoviruses + PLV_BS13 | 0 | 0.000 | 99.243 |
| Others | 212 | 0.757 | 100 |
