# Supplementary material for: A billion years arms-race between viruses, virophages, and eukaryotes
Source: eLife. 2023 Jun 26;12:RP86617. doi: 10.7554/eLife.86617 (PMC10328495; doi:10.7554/eLife.86617)
Supplement: Supplementary file 3. [file elife-86617-supp3.docx]

**Supplementary file 3.** Cytoplasmic linear plasmids used for querying the databases in search for protein homologues.

| **Plasmid** | **GenBank accession** | **Host organism** (Order Saccharomycetales) |
| --- | --- | --- |
| pDH4C | MF795093.1 | *Debaryomyces hansenii* (Debaryomycetaceae) |
| pGK12 | X07776.1 | *Kluyveromyces lactis* (Saccharomycetaceae) |
| pKPGS115 | CP014724.1 | *Komagataella phaffii* (Phaffomycetaceae) |
| pKP | CP014714.1 | *Komagataella phaffii* (Phaffomycetaceae) |
| pKPCBS743 | MG491503.1 | *Komagataella phaffii* (Phaffomycetaceae) |
| pPac1-1 | AM180622.1 | *Millerozyma acacia* (Debaryomycetaceae) |
| pPE1B | AJ278986.2 | *Schwanniomyces etchellsi* (Debaryomycetaceae) |
| pSKL | X54850.1 | *Lachancea kluyveri* (Saccharomycetaceae) |
